# Associations of Health-Related Physical Fitness with Waist Circumference and Abdominal Obesity Risk in Taiwanese Adults

**DOI:** 10.3390/healthcare10071164

**Published:** 2022-06-22

**Authors:** Yun-Tsung Chen, Po-Fu Lee, Chi-Fang Lin, Yan-Jhu Su, Hui-Ling Chen, Pin-Chun Chen, Hsueh-Yi Lin, Chien-Chang Ho

**Affiliations:** 1 Department of Physical Education and Sport Sciences, National Taiwan Normal University, Taipei City 106, Taiwan; cthero178@hotmail.com (Y.-T.C.); aa830521@gmail.com (C.-F.L.); 2 Department of Leisure Industry and Health Promotion, National Ilan University, Yilan County 260, Taiwan; pflee@niu.edu.tw (P.-F.L.); hilin@niu.edu.tw (H.-Y.L.); 3 College of Humanities and Management, National Ilan University, Yilan County 260, Taiwan; 4 Department of Gerontology, University of Massachusetts Boston, Boston, MA 02125, USA; yanjhu.su001@umb.edu; 5 Graduate Institute of Educational Leadership and Development, Fu Jen Catholic University, New Taipei City 242, Taiwan; 024145@mail.fju.edu.tw; 6 Graduate Institute of Sports Training, University of Taipei, Taipei City 111, Taiwan; nana231688@gmail.com; 7 Department of Physical Education, Fu Jen Catholic University, New Taipei City 24205, Taiwan; 8 Research and Development Center for Physical Education, Health and Information Technology, College of Education, Fu Jen Catholic University, New Taipei City 24205, Taiwan; 9 Office of Physical Education, Fu Jen Catholic University, New Taipei City 24205, Taiwan

**Keywords:** physical fitness, waist circumference, adiposity, adults

## Abstract

In this study, we aimed to determine the associations among health-related physical fitness measurements, waist circumference (WC), and abdominal obesity risk in Taiwanese adults. We conducted a cross-sectional study based on data from the 2017 Taiwan Scientific Physical Fitness Survey (the TSPFS). We collected the responses from 17,973 participants (7907 men and 10,066 women) aged 23–64 years for this study. The participants completed the study protocol with a standardized structural questionnaire and a series of health-related physical fitness measurements. The fitness measurements included cardiorespiratory fitness (measured by a 3-min progressive knee-up and step test), muscular fitness (measured by a hand grip strength test), and flexibility (measured by a sit-and-reach test). Our anthropometric measurements included height, weight, WC, hip circumference (HC), body mass index (BMI), and waist-to-hip ratio (WHR). We identified the quartiles of the health-related physical fitness results as the dependent variable in the multiple linear and multiple logistic regression analysis to determine the associations of the physical fitness measurements with WC distribution and abdominal obesity risk. We also considered the dose–response relationship. We found that cardiorespiratory fitness, relative grip strength, and flexibility were each significantly negatively associated with WC, but absolute grip strength was significantly positively associated with WC. We also found that higher levels of cardiorespiratory fitness, relative grip strength, and flexibility were each associated with a lower risk of abdominal obesity. Moreover, our secondary finding was of a dose–response relationship between physical fitness performance, WC, and abdominal obesity risk. In short, health-related physical fitness was an effective predictor of waist circumference for both sexes of Taiwanese adults, and higher levels of health-related physical fitness were associated with lower risks of abdominal obesity in Taiwanese adults.

## 1. Introduction

Obesity is a global public health and economic problem. In 2016, more than 650 million adults (approximately 13% of the global adult population) were classified as obese [1]. In 2017, 4.7 million deaths worldwide were attributable to obesity [2]. Furthermore, obesity resulted in a global economic burden of USD 2 trillion, which was 2.8% of the global gross domestic product (GDP) [3]. In 2018, 17.2% of Taiwanese adults were classified as obese [4]. In 2020, the levels of abdominal obesity in Taiwanese adults aged 19 years and older were 47% for men and 53% for women [5]. Accordingly, successful weight management is an important issue in Taiwan.

Obesity, especially abdominal obesity (e.g., excessive visceral and subcutaneous adipose tissue) is a major risk factor for metabolic syndrome, diabetes, disease (CVD), and premature mortality [6,7]. Waist circumference (WC) is considered a good indicator for central obesity [8]. WC is a more powerful predictor than the body mass index (BMI) of the future risk of type 2 diabetes, CVD, and metabolic syndrome [6,9,10]. In addition, the levels of health-related physical fitness are common predictors of obesity-associated health risks [11].

Health-related physical fitness consists of cardiorespiratory fitness, muscular fitness (including muscle strength and endurance), body composition, and flexibility, each of which influences some aspect of health [12]. For example, higher levels of cardiorespiratory fitness are associated with lower risks of chronic diseases, such as hypertension, dyslipidemia, insulin resistance, and CVD [11]. Greater muscle strength is associated with lower risks of osteoporosis, disability, metabolic syndrome, and CVD events [11]. In addition, flexibility has beneficial effects on joint range of motion, postural stability, and balance and may reduce the risk of muscle injury and falls in elderly adults [13,14,15].

The 3-min progressive knee-up and step (3MPKS) test is a safe and valid method for predicting the maximal oxygen uptake, which is the main criterion for measuring cardiorespiratory fitness [16]. This test does not require a step-up box, expensive equipment, or exercising to exhaustion; accordingly, older adults may be more willing to participate in 3MPKS tests rather than the alternatives to reduce the risk of falls and to address the risks associated with obesity [16].

On the other hand, studies pointed out that health-related physical fitness is associated with abdominal obesity when using WC as its measurement. For example, cardiorespiratory fitness is negatively associated with the BMI and WC [6,17,18,19,20]. Moreover, higher levels of abdominal obesity have been associated with lower muscular fitness in the trunk and lower extremities, as determined by a 1-min sit-up test and vertical jump performance [6,18]. However, to the best of our knowledge, the relationship between 3MPKS testing, WC, and abdominal obesity remains unclear and warrants further investigation. Moreover, little is known about the relationship between abdominal obesity with other health-related physical fitness measurements, such as muscle strength and flexibility. In addition, relatively limited information is available regarding the relationship between abdominal obesity and upper extremity muscle strength (e.g., grip strength).

Therefore, our aims in this study were to investigate the association of health-related physical fitness measurements with the WC and abdominal obesity risk in Taiwanese adults and to examine whether a dose–response relationship exists between the physical fitness performance, WC, and abdominal obesity risk.

## 2. Materials and Methods

### 2.1. Study Participants, Design, and Procedure

We obtained the analytic data for this study from the Taiwan Scientific Physical Fitness Survey (TSPFS), which was conducted by the Taiwan Sport Administration, Ministry of Education. Using technology, the TSPFS was designed to evaluate the health-related physical fitness components in adults, including cardiorespiratory endurance, muscular strength and endurance, flexibility, and body composition.

We included 17,973 Taiwanese nationals (7907 men and 10,066 women) between the ages of 23 and 64 who participated in the TSPFS from September 2017 to November 2017. We excluded potential participants who had systolic blood pressure ≥ 140 mmHg, diastolic blood pressure ≥ 90 mmHg, heart disease, hypertension, chest pain, vertigo, and/or musculoskeletal disorders.

Our study was based on convenience sampling at 18 examination stations in Taiwan. All the participants had been previously informed by the Sport Administration of the objectives and procedures of the TSPFS survey, and all the participants provided their informed consent. A detailed study protocol for the TSPFS is available at https://isports.sa. gov.tw/index.aspx (accessed on 9 June 2022).

We conducted this study in accordance with the Declaration of Helsinki, and the protocol was approved by the Institutional Review Board of Fu Jen Catholic University in Taiwan (FJU-IRB C110113).

### 2.2. Data Collection Procedures

We collected the participants’ sociodemographic characteristics, such as age, sex, education level, occupational condition, monthly income, marital status, and relationship status, through face-to-face interviews using a structured questionnaire that had been previously validated and published [20]. We categorized education into three levels: elementary school or lower, junior or senior school, and college/university or higher. We determined occupational condition based on employment status, including the subcategories of currently employed, unemployed, and other. We categorized monthly income as ≤NTD 20,000, NTD 20,001–40,000, or ≥NTD 40,001. We also categorized marital status into three subgroups: never married, married, or divorced/separation/widowed. We subcategorized relationship status as living or not living with someone else.

### 2.3. Anthropometric Variables and Definition of Abdominal Obesity

Trained examiners obtained the TSPFS’s anthropometric measurements, including height, weight, WC, and hip circumference (HC), in accordance with a standardized protocol [6,20,21]. During the TSPFS, the participants were instructed to stand upright without shoes or heavy clothes, with their heels touching the wall and with a straight body facing forward. Height was measured to the nearest 1 cm using a stadiometer. We measured body weight to the nearest 1 kg using an electronic weightometer. We calculated the BMI as body weight in kilograms divided by height in meters squared (kg/m^2^). We performed the WC measurements (measured to the nearest 1 cm) twice at midway between the lowest rib crest after normal exhalation, and the mean value was recorded. We measured the HC (to the nearest 1 cm) twice at the site of the largest convexity of the buttocks below the hip plates, and recorded the mean value. We derived the waist-to-hip ratio (WHR) from the ratio between the WC and HC. We defined abdominal obesity according to the guidelines of the International Diabetes Federation (IDF) for Asian populations: WC ≥ 90 cm for men and ≥80 cm for women [22].

### 2.4. Health-Related Physical Fitness Measures

We conducted the following tests to determine health-related physical fitness: cardiorespiratory fitness via the 3MPKS test (mL/kg/min) [16,23], muscular fitness via hand grip strength (kg) [24], and flexibility via the sit-and-reach test (cm) [25]. We measured the hand grip strength with an electronic hand grip dynamometer; we determined the average of a participant’s two dominant hand grip attempts. We conducted the sit-and-reach test twice with a sit-and-reach box and a measuring scale; we marked the level of the feet at 30 cm and determined the average distance from a participant’s two attempts.

We asked participants not to participate in any other moderate- or vigorous-intensity physical activity before the tests and to perform a 10-min warm-up that was led by the examiner before the scientifically measured physical fitness assessment. All participants performed the tests in the following order with a break period (3–5 min) between tests: hand grip strength, sit-and-reach, and 3MPKS tests.

### 2.5. Statistical Analysis

We conducted all statistical analyses using SAS software version 9.4 (SAS Institute, Cary, NC, USA). We conducted Student’s *t*-tests and chi-square tests to estimate the differences in the demographic characteristics, anthropometric variables, and health-related physical fitness measurements, including abdominal obesity status. With scientific physical fitness measurements as the dependent variable, we used multiple linear regression to examine the associations between health-related physical fitness measurements and WC.

To examine the dose–response relationship of a health-related physical fitness performance with the WC and abdominal obesity risk, we established four different categories (quartiles) for each health-related physical fitness measurement. The lowest quartile (the reference group) comprised participants who performed the best in each health-related physical fitness measurement.

We conducted unconditional logistic regression analyses to evaluate the linear association between cardiorespiratory fitness, muscle fitness or flexibility, and abdominal obesity risks. We adjusted all regression models for age, education, occupational condition, monthly income, marital status, relationship status, and other health-related physical fitness measurements. In addition, we calculated the adjusted odds ratios (ORs) with 95% confidence intervals (CIs). We expressed all data as means ± standard deviation or frequency (percentage). We established the significance level at *p* < 0.05.

## 3. Results

As noted, we included 17,973 participants (7907 men and 10,066 women) with complete data that we obtained from the TSPFS. Table 1 shows the participants’ demographic characteristics and anthropometric variables. We divided the participants into dichotomous groups—abdominal obesity and nonabdominal obesity—for each sex; 80% of the participants (79.84%) were nonabdominally obese. We determined statistically significant differences between the abdominal obesity group and the nonabdominal obesity group (*p* < 0.001) for all relevant variables, including age, height, body weight, BMI, WC, HC, WHR, education level, employment status, income level, marital status, and relationship status.

Table 2 shows the health-related physical fitness measurements according to the abdominal obesity status of the participants. The statistically significant differences between the abdominal obesity group and the nonabdominal obesity group (*p* < 0.05) are shown for all the relevant variables (3MPKS, grip strength, grip strength/body weight, and sit-and-reach tests). Notably, the *p*-value of grip strength for men in the abdominal obesity group and the nonabdominal obesity group was close to our typical rejection level at 0.05 (*p* = 0.048). The *p*-values of all the other variables were less than 0.001 (*p* < 0.0001).

Determining the linear association between the health-related physical fitness measurements and WC.

Table 3 shows the regression coefficients we used for predicting the WC using different health-related physical fitness measurements. This table contains two models: an unadjusted model and a model adjusted for potential confounders (age, education, occupation, monthly income, marital status, relationship status, and other physical fitness measurements). The results showed statistically significant differences between the abdominal obesity group and the nonabdominal obesity group (*p* < 0.0001) for all relevant variables (3MPKS, grip strength, grip strength/body weight, and sit-and-reach tests) in both the unadjusted and adjusted groups.

Determining the dose–response relationship between the health-related physical fitness measurements and WC.

Table 4 shows the regression coefficients we used for predicting the WC using different quartiles of health-related physical fitness measurements. This table also contains two models: an unadjusted model and a model adjusted for potential confounders, as above. We use the fourth quartile as a reference group for each variable in this table. For men, most quartiles of the health-related physical measurements were statistically significant (*p* < 0.0001) in both the unadjusted and adjusted models, except for the third quartile of the sit-and-reach test in both the unadjusted and the adjusted models (*p* = 0.713 and *p* = 0.879, respectively), which did not meet our typical rejection level of 0.05. In addition, although the second quartile of the sit-and-reach test in both the unadjusted and the adjusted models (*p* = 0.020 and *p* = 0.012, respectively) met our typical rejection level of 0.05, the levels were not less than 0.01. For women, all the quartiles of the health-related physical measurements were statistically significant (*p* < 0.0001) in both the unadjusted and the adjusted models.

Determining the dose–response relationship between the health-related physical fitness measurements and abdominal obesity risk.

Table 5 shows the multivariate adjusted ORs for abdominal obesity in relation to the quartiles of the physical fitness measurements. This table also contains two models: an unadjusted model and a model adjusted for potential confounders, as above. We use the fourth quartile as a reference group for each variable in this table. For men, most quartiles of the health-related physical measurements were statistically significant (*p* < 0.0001) in both the unadjusted and adjusted models. However, for the second quartile of the sit-and-reach test among men, the unadjusted (OR = 1.087, *p* = 0.362) and adjusted models (OR = 1.157, *p* = 0.119) did not meet our typical rejection level of 0.05. Moreover, for the third quartile of the sit-and-reach test among men, the unadjusted (OR = 1.110, *p* = 0.256) and adjusted models (OR = 1.118, *p* = 0.235) did not meet our typical rejection level of 0.05. For women, all the quartiles of the health-related physical measurements were statistically significant (*p* < 0.0001) in both the unadjusted and adjusted models. Notably, after adjusting for potential confounders, the *p*-value of the third quartile for grip strength/body weight among women was less in the adjusted than in the unadjusted model (OR = 1.231, *p* < 0.009; OR = 1.342, *p* < 0.0001, respectively).

## 4. Discussion

In this cross-sectional study, we investigated the relationships among the health-related physical fitness measurements, WC, and abdominal obesity risk after adjusting for possible confounding factors. Our main findings in this study were as follows: (1) the 3MPKS, relative grip strength, and sit-and-reach tests were effective in providing predictors of the WC among men and women, and (2) higher levels of cardiorespiratory fitness, relative grip strength, and flexibility were each associated with lower risks of abdominal obesity. We also reached the secondary finding, set out above, about the dose–response relationships among the health-related physical fitness levels, WC, and abdominal obesity risk.

We found that, in 2017, the prevalence of abdominal obesity for Taiwanese adults between the ages of 23 and 64 years was 20%; they had a higher WC, waist-to-hip ratio, body weight, and BMI than Taiwanese adults outside that age group. In addition, both men and women in the nonabdominal obesity group exhibited a higher performance in the 3MPKS, relative grip strength, and sit-and-reach tests than both men and women in the abdominal obesity group.

However, a 2020 survey indicated that the mean BMI for men and women was 25.3 kg/m and 23.8 kg/m, respectively, and that ≥40% of Taiwanese adults were classified as abdominally obese at age 19 years and older [5]. Therefore, successful weight management is an important issue for individuals in Taiwan, with the goals of reducing the risk of abdominal obesity and improving their health-related physical fitness.

To the best of our knowledge, this is the first study to report that the 3MPKS test performance was significantly and negatively associated with the WC and abdominal obesity risk among Taiwanese men and women. Our findings showed that cardiorespiratory fitness (measured by a maximal exercise test, submaximal cycle ergometer, and six-minute walk test) was negatively associated with the WC in both sexes [17,18,26,27]. The findings of a similar study indicated that the 3MPKS test (with a 20.3-cm step height and a pace of 24 steps/min) performance was negatively associated with abdominal adiposity in both men and women. However, Lee et al. found that the results of a 3MPKS test with a 35-cm step height and a pace of 24 steps/min was only negatively associated with abdominal obesity in men [6]. The differences in the step heights during the 3MPKS tests may be the reason for this disparity.

Furthermore, compared with tests requiring a step-up box and expensive metabolic equipment, the 3MPKS test is a valid, time-efficient, and more convenient method for predicting cardiorespiratory fitness [16]. Future researchers should investigate whether an optimal step height exists for the 3MPKS test to measure cardiorespiratory fitness in people of different sexes, ages, and obesity status.

The WC has been negatively associated with muscle endurance performance as measured via 1-min sit-ups and push-ups but not with isometric muscle strength performance (absolute grip strength) [6,18,28]. In the current study, we observed that the absolute grip strength was positively associated with the WC (men, β = 0.12; women, β = 0.26), whereas the relative grip strength (based on hand grip data that were normalized according to body weight) was negatively associated, in both sexes, with both WC (men, β = −19.60; women, β = −14.96) and abdominal obesity risk. Grip strength is a good predictor of total muscle strength [29], which is positively associated with physical performance when measured by a 6-m timed up-and-go test and a 3-m walk test [30]. The results of a similar study indicated that the relative grip strength was negatively associated with WC in young adults [31]. In addition, the relative grip strength is more strongly associated than absolute grip strength with the metabolic syndrome and CVD risk factors [32,33]. Therefore, we suggest that the relative grip strength (β = −15 ~ −20) may be a more accurate predictor than the absolute grip strength of the WC (β = 0.1~0.3), 3MPKS (β = −0.4), and sit-and-reach test (β = −0.1) performances. Further investigation is needed to examine the relationships between the relative muscle strength and risks of abdominal obesity in adolescents and older adults.

Flexibility has beneficial effects on the joint ranges of motion, static and dynamic balance, and muscle tightness release. Flexibility may reduce the risk of musculoskeletal injury and falling in young and elderly adults [14,34]. In this study, we observed that flexibility was significantly and negatively associated with both the WC and abdominal obesity risk in both men and women. The findings of a similar study indicated that flexibility negatively correlated with the WC and positively correlated with high-density lipoproteins in both sexes [5,34]. Moreover, Chang et al. suggested that the metabolic syndrome was associated with a decreased flexibility in elderly adults [35].

The present study had some limitations. First, our study had a cross-sectional design study; thus, we could not explore the causal relationship of the changes in health-related physical fitness and the development of abdominal obesity. Second, the subjects were healthy Taiwanese adults aged 23–64 years who volunteered for a series of health-related physical fitness examinations; whether the results can be generalized to persons outside this group requires further study.

## 5. Conclusions

Our findings demonstrated that adults with abdominal obesity had higher WC, waist-to-hip ratios, body weights, and BMI than the other adults. The 3MPKS, relative grip strength, and sit-and-reach tests were each good predictors of the WC. Higher levels of health-related physical fitness were associated with lower WC and lower abdominal obesity risks. We observed dose–response relationships in the aforementioned variables.

## Figures and Tables

**Table 1:** Characteristics of the study participants, with or without abdominal obesity.

Variable	Men (N = 7907)			Women (N = 10,066)		
	AO (*n* = 1238)	Non-AO (*n* = 6669)	*p*	AO (*n* = 2385)	Non-AO (*n* = 7681)	*p*
Age (years)	41.37 ± 11.14	36.65 ± 10.94	<0.0001 *	38.78 ± 12.49	37.09 ± 10.63	<0.0001 *
Height (cm)	172.95 ± 6.68	171.40 ± 6.34	<0.0001 *	160.70 ± 6.65	159.50 ± 5.50	<0.0001 *
Body weight (kg)	83.87 ± 9.94	70.10 ± 9.85	<0.0001 *	64.93 ± 10.19	54.34 ± 6.84	<0.0001 *
BMI (kg/m^2^)	28.02 ± 2.74	23.84 ± 2.95	<0.0001 *	25.14 ± 3.63	21.36 ± 2.43	<0.0001 *
WC (cm)	95.72 ± 5.27	79.54 ± 6.60	<0.0001 *	86.55 ± 5.85	71.45 ± 5.60	<0.0001 *
HC (cm)	104.94 ± 5.62	95.74 ± 7.25	<0.0001 *	93.08 ± 8.17	90.53 ± 6.61	<0.0001 *
WHR	0.91 ± 0.05	0.83 ± 0.04	<0.0001 *	0.86 ± 0.06	0.79 ± 0.04	<0.0001 *
Elementary school or lower	1.5	0.4		3.6	0.7	
Junior or senior school	17.6	7.6		21.8	9.0	
College or higher	80.9	92.0		74.6	90.3	
Currently employed (%)			<0.0001 *			<0.0001 *
Yes	88.7	90.0		77.7	87.5	
No	6.3	7.3		18.5	10.2	
Other	5.0	2.7		3.8	2.3	
Income level (%)			<0.0001 *			<0.0001 *
≤NTD 20,000	8.6	8.2		19.3	10.0	
NTD 20,001–40,000	30.6	23.6		40.0	35.6	
≥NTD 40,001	60.8	68.2		40.7	54.4	
Marital status (%)			<0.0001 *			<0.0001 *
Never married	31.7	44.1		36.0	41.8	
Married	65.7	49.6		58.4	51.8	
Divorced/separation/widowed	2.7	6.3		5.6	6.4	
Relationship status (%)			<0.0001 *			<0.0001 *
Living with someone	90.8	78.8		89.6	84.5	
Not living with someone	9.2	21.2		10.4	15.5	

Abbreviations: AO, abdominal obesity; BMI, body mass index; HC, hip circumference; NTD, New Taiwan Dollar; WC, waist circumference; and WHR, waist-to-hip ratio. Values are expressed as the means ± SD. Abdominal obesity, WC ≥ 90/80 cm; nonabdominal obesity, WC < 80/90 cm. * *p* < 0.05.

**Table 2:** Health-related physical fitness measurements according to the abdominal obesity status.

Variable	Men (N = 7907)			Women (N = 10,066)		
	AO (*n* = 1238)	Non-AO (*n* = 6669)	*p*	AO (*n* = 2385)	Non-AO (*n* = 7681)	*p*
3MPKS (mL/kg/min)	39.91 ± 4.07	42.26 ± 5.29	<0.0001 *	33.00 ± 4.84	35.21 ± 5.14	<0.0001 *
Grip strength (kg)	41.96 ± 9.09	41.41 ± 8.91	0.048 *	26.64 ± 6.82	25.17 ± 5.48	<0.0001 *
Grip strength/BW	0.50 ± 0.11	0.60 ± 0.13	<0.0001 *	0.42 ± 0.11	0.47 ± 0.10	<0.0001 *
Sit-and-reach test (cm)	20.29 ± 9.89	21.69 ± 9.70	<0.0001 *	26.10 ± 9.80	27.66 ± 10.58	<0.0001 *

Abbreviations: 3MPKS, 3-min progressive knee-up and step; AO, abdominal obesity; BW, body weight; SD, standard deviation; and WC, waist circumference. Values are expressed as the means ± SD. Abdominal obesity, WC ≥ 90/80 cm; nonabdominal obesity, WC < 80/90 cm. * *p* < 0.05.

**Table 3:** Regression coefficients for predicting waist circumference using different health-related physical fitness measurements.

Variable	Model 1 (Unadjusted)			Model 2 (Adjusted ^a^)		
	*β*	SE	*p*	*β*	SE	*p*
**Men (N = 7907)**						
3MPKS (mL/kg/min)	−0.399	0.019	<0.0001 *	−0.358	0.023	<0.0001 *
Grip strength (kg)	0.124	0.011	<0.0001 *	0.121	0.011	<0.0001 *
Grip strength/BW	−17.629	0.752	<0.0001 *	−19.596	0.761	<0.0001 *
Sit-and-reach test (cm)	−0.068	0.010	<0.0001 *	−0.067	0.010	<0.0001 *
**Women (N = 10,063)**						
3MPKS (ml/kg/min)	−0.410	0.016	<0.0001 *	−0.421	0.019	<0.0001 *
Grip strength (kg)	0.257	0.014	<0.0001 *	0.264	0.014	<0.0001 *
Grip strength/BW	−15.173	0.797	<0.0001 *	−14.959	0.793	<0.0001 *
Sit-and-reach test (cm)	−0.069	0.008	<0.0001 *	−0.068	0.008	<0.0001 *

Abbreviations: 3MPKS, 3-min progressive knee-up and step; BW, body weight; SE, standard error; and β, regression coefficient. * *p* < 0.05. ^a^ Adjusted for age, education, occupation, monthly income, marital status, relationship status, and other physical fitness measurements.

**Table 4:** Regression coefficients for predicting the waist circumference using different quartiles of health-related physical fitness measurements.

Variables	Model 1 (Unadjusted)			Model 2 (Adjusted ^a^)		
	*β*	SE	*p*	*β*	SE	*p*
**Men (N = 7907)**						
3MPKS (mL/kg/min)						
<38.25 (Q1)	5.011	0.274	<0.0001 *	3.820	0.330	<0.0001 *
38.25–41.65 (Q2)	4.800	0.270	<0.0001 *	4.265	0.291	<0.0001 *
41.66–45.55 (Q3)	4.137	0.269	<0.0001 *	3.864	0.273	<0.0001 *
>45.55 (Q4)	Ref.	—	—	Ref.	—	—
Test for trend	*p* < 0.0001 *			*p* < 0.0001 *		
Grip strength/BW						
<0.50 (Q1)	6.130	0.279	<0.0001 *	6.631	0.279	<0.0001 *
0.50–0.58 (Q2)	4.856	0.270	<0.0001 *	5.030	0.266	<0.0001 *
0.59–0.67 (Q3)	3.304	0.265	<0.0001 *	3.376	0.261	<0.0001 *
>0.67 (Q4)	Ref.	—	—	Ref.	—	—
Test for trend	*p* < 0.0001 *			*p* < 0.0001 *		
Sit-and-reach (cm)						
<14.50 (Q1)	1.451	0.272	<0.0001 *	1.386	0.270	<0.0001 *
14.50–21.00 (Q2)	0.619	0.267	0.020 *	0.666	0.265	0.012 *
21.01–28.00 (Q3)	0.099	0.269	0.713	0.041	0.267	0.879
>28.00 (Q4)	Ref.	—	—	Ref.	—	—
Test for trend	*p* < 0.0001 *			*p* < 0.0001 *		
**Women (N = 10,063)**						
3MPKS (mL/kg/min)						
<31.17 (Q1)	5.248	0.234	<0.0001 *	5.259	0.270	<0.0001 *
31.17–34.42 (Q2)	3.902	0.232	<0.0001 *	4.070	0.242	<0.0001 *
34.43–37.97 (Q3)	2.162	0.232	<0.0001 *	2.321	0.233	<0.0001 *
>37.97 (Q4)	Ref.	—	—	Ref.	—	—
Test for trend	*p* < 0.0001 *			*p* < 0.0001 *		
Grip strength/BW						
<0.39 (Q1)	4.484	0.241	<0.0001 *	4.352	0.240	<0.0001 *
0.39–0.45 (Q2)	2.043	0.235	<0.0001 *	2.110	0.233	<0.0001 *
0.46–0.52 (Q3)	1.036	0.234	<0.0001 *	1.153	0.232	<0.0001 *
>0.52 (Q4)	Ref.	—	—	Ref.	—	—
Test for trend	*p* < 0.0001 *			*p* < 0.0001 *		
Sit-and-reach test (cm)						
<20.00 (Q1)	1.726	0.236	<0.0001 *	1.704	0.233	<0.0001 *
20.00–27.00 (Q2)	1.667	0.231	<0.0001 *	1.606	0.228	<0.0001 *
27.01–34.50 (Q3)	1.019	0.233	<0.0001 *	0.914	0.230	<0.0001 *
>34.50 (Q4)	Ref.	—	—	Ref.	—	—
Test for trend	*p* < 0.0001 *			*p* < 0.0001 *		

Abbreviations: 3MPKS, 3-min progressive knee-up and step; BW, body weight; SE, standard error; and β, regression coefficient. * *p* < 0.05. ^a^ Adjusted for age, education, occupation, monthly income, marital status, relationship status, and other physical fitness measurements.

**Table 5:** Multivariate adjusted ORs for abdominal obesity in relation to the quartiles of the physical fitness measurements after adjustment for potential confounders.

Variables	Model 1 (Unadjusted)			Model 2 (Adjusted ^a^)		
	OR	95% CI	*p*	OR	95% CI	*p*
**Men (N = 7907)**						
3MPKS (mL/kg/min)						
<38.25 (Q1)	6.199	4.881–7.874	<0.0001 *	4.416	3.367–5.792	<0.0001 *
38.25–41.65 (Q2)	5.047	3.972–6.414	<0.0001 *	4.421	3.427–5.704	<0.0001 *
41.66–45.55 (Q3)	4.310	3.385–5.487	<0.0001 *	4.207	3.282–5.392	<0.0001 *
>45.55 (Q4)	Ref.	—	—	Ref.	—	—
Test for trend	*p* < 0.0001 *			*p* < 0.0001 *		
Grip strength/BW						
<0.50 (Q1)	9.081	6.850–12.038	<0.0001 *	10.060	7.549–13.405	<0.0001 *
0.50–0.58 (Q2)	5.800	4.363–7.709	<0.0001 *	6.369	4.779–8.488	<0.0001 *
0.59–0.67 (Q3)	3.329	2.475–4.478	<0.0001 *	3.552	2.635–4.789	<0.0001 *
>0.67 (Q4)	Ref.	—	—	Ref.	—	—
Test for trend	*p* < 0.0001 *			*p* < 0.0001 *		
Sit-and-reach (cm)						
<14.50 (Q1)	1.349	1.131–1.610	0.001 *	1.435	1.198–1.719	<0.0001 *
14.50–21.00 (Q2)	1.087	0.908–1.301	0.362	1.157	0.963–1.391	0.119
21.01–28.00 (Q3)	1.110	0.927–1.330	0.256	1.118	0.930–1.344	0.235
>28.00 (Q4)	Ref.	—	—	Ref.	—	—
Test for trend	*p* = 0.002 *			*p* < 0.0001 *		
**Women (N = 10,063)**						
3MPKS (mL/kg/min)						
<31.17 (Q1)	3.585	3.110–4.133	<0.0001 *	3.508	2.976–4.136	<0.0001 *
31.17–34.42 (Q2)	2.313	2.002–2.672	<0.0001 *	2.396	2.057–2.792	<0.0001 *
34.43–37.97 (Q3)	1.406	1.207–1.636	<0.0001 *	1.456	1.246–1.702	<0.0001 *
>37.97 (Q4)	Ref.	—	—	Ref.	—	—
Test for trend	*p* < 0.0001 *			*p* < 0.0001 *		
Grip strength/BW						
<0.39 (Q1)	3.153	2.729–3.642	<0.0001 *	3.260	2.812–3.781	<0.0001 *
0.39–0.45 (Q2)	1.621	1.397–1.882	<0.0001 *	1.759	1.509–2.050	<0.0001 *
0.46–0.52 (Q3)	1.231	1.054–1.438	0.009 *	1.342	1.145–1.573	<0.0001 *
>0.52 (Q4)	Ref.	—	—	Ref.	—	—
Test for trend	*p* < 0.0001 *			*p* < 0.0001 *		
Sit-and-reach (cm)						
<20.00 (Q1)	1.498	1.302–1.723	<0.0001 *	1.507	1.306–1.739	<0.0001 *
20.00–27.00 (Q2)	1.596	1.392–1.830	<0.0001 *	1.590	1.383–1.829	<0.0001 *
27.01–34.50 (Q3)	1.375	1.196–1.581	<0.0001 *	1.353	1.173–1.560	<0.0001 *
>34.50 (Q4)	Ref.	—	—	Ref.	—	—
Test for trend	*p* < 0.0001 *			*p* < 0.0001 *		

Abbreviations: 3MPKS, 3-min progressive knee-up and step; BW, body weight; CI, confidence interval; and OR, odds ratio. * *p* < 0.05. ^a^ Adjusted for age, education, occupation, monthly income, marital status, relationship status, and other physical fitness measurements.

## Data Availability

The data that support the findings of this study are available from the Sports Cloud: Information and Application Research Center of Sports for All, Sport Administration, Ministry of Education, Taiwan. However, restrictions apply to the availability of these data, as they were used under license for the current study. The data are not publicly available but are available from the authors upon reasonable request and with permission from the Sports Cloud: Information and Application Research Center of Sports for All, Sport Administration, Ministry of Education, Taiwan.
